# Lung Infection—A Public Health Priority

**DOI:** 10.1371/journal.pmed.0030076

**Published:** 2006-01-17

**Authors:** Joseph P Mizgerd

## Abstract

The pervasive burden of lung infections receives proportionately little attention from the biomedical and public health communities, argues Mizgerd.

Emerging lung infections capture the world's imagination because of the potential for pandemics. Recent examples include avian influenza and the severe acute respiratory syndrome (SARS). However, even in the absence of new pathogens or pandemics, lung infections have tremendous impact. Lung infections cause more disease than better-recognized threats to the public's health such as cancer, heart attacks, strokes, HIV/AIDS, tuberculosis, or malaria. This persistent and pervasive burden of lung infections receives proportionately little attention from the biomedical and public health communities.

## The Global Burden of Lung Infections

The Burden of Disease Project [1] at the World Health Organization (WHO) collects statistics that can be used to determine the public health impact of different diseases. The metric of disability-adjusted life years (DALYs) lost takes into account the amount of otherwise healthy life lost to morbidity and/or mortality. Diseases were categorized according to the International Classification of Diseases (ICD) from WHO [2].

The “lung infections” category includes “Influenza and pneumonia” (ICD–10 codes J10–18) and “Other acute lower respiratory infections” (ICD–10 J20–22), but it excludes “Tuberculosis” (ICD–10 A15–19) and “HIV disease resulting in infectious and parasitic diseases” (ICD–10 B20). By excluding respiratory tuberculosis as well as pneumonias in patients with HIV/AIDS, these statistics might be considered by some to underestimate the burden of disease due to lung infections. Even with those exclusions, lung infections accounted for more than 6% of the total global burden of disease in 2002. This disease burden is greater than that of other better-recognized causes of disease (Figure 1). This impressive burden is not an anomaly of that particular year, due to SARS or any other unusual epidemic or event, but is instead the norm. Since 1990, when WHO began compiling and presenting such statistics, lung infections have consistently caused more burden than any of the diseases identified in Figure 1 [3,4].

**Figure 1:**
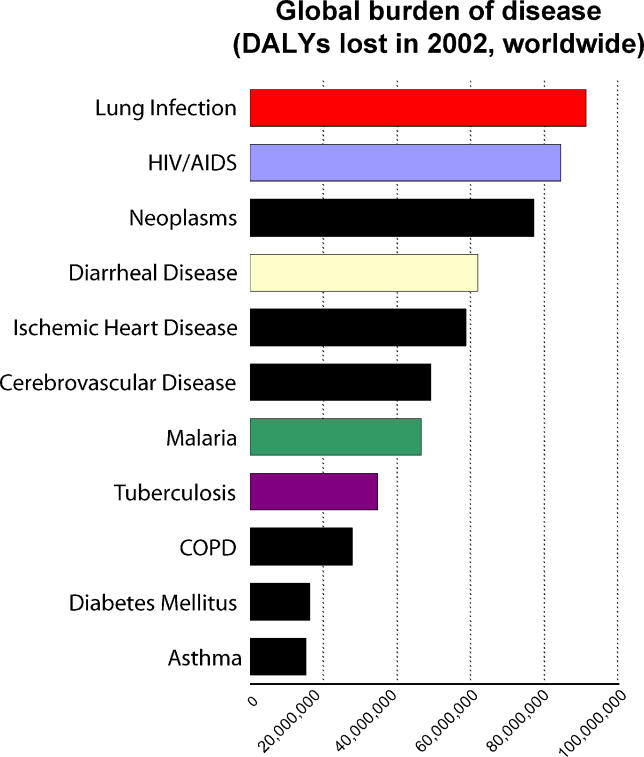
The Global Burden of Selected Diseases in 2002, as Measured by Disability-Adjusted Life Years (DALYs) Lost Worldwide COPD, chronic obstructive pulmonary disease

### The Disproportionate Burden on the Poor

Lung infections are especially common and severe among the poor. When the relative burden of disease in communities is assessed by normalizing DALYs to population size, lung infections caused the loss of 2,983 DALYs/100,000 population in the poorest regions compared to 137 DALYs/100,000 population in the wealthiest. Thus, poverty is associated with a more than 20-fold increase in the relative burden of lung infections.

According to WHO, 2.6 billion people live in “Low Income” countries, with Gross National Income (GNI) per capita ≤US$765, and 2.2 billion people live in “Lower Middle Income” countries, with GNI per capita of US$766–US$3,035. This single incremental improvement in income is associated with a dramatic difference in the relative burden of disease caused by lung infections. Lung infections caused the loss of 535 DALYs per 100,000 population in the “Lower Middle Income” population in 2002. Comparing this figure to the relevant figures listed above, it may be surmised that the vast majority (86%) of the difference due to income occurs between the lowest income group and the next-to-lowest income group. Thus, the poor are especially likely to suffer from lung infections, and relatively modest income improvements may substantially lessen their burden of lung infection. As with many infectious diseases [5], fighting poverty and improving health care for the impoverished will greatly decrease the global burden of lung infections.

## Lung Infections Threaten All Economic Groups

Among those who live in wealthy societies, infectious diseases cause less of a burden than do chronic diseases such as cancers and cardiovascular diseases. However, even among advantaged populations, lung infections are remarkably prominent. In the wealthiest as well as the poorest regions of the world, lung infections cause a greater burden than any other infectious disease (Figure 2). Thus, while climbing the socioeconomic ladder associates with a lesser burden, lung infections threaten across the economic spectra.

**Figure 2:**
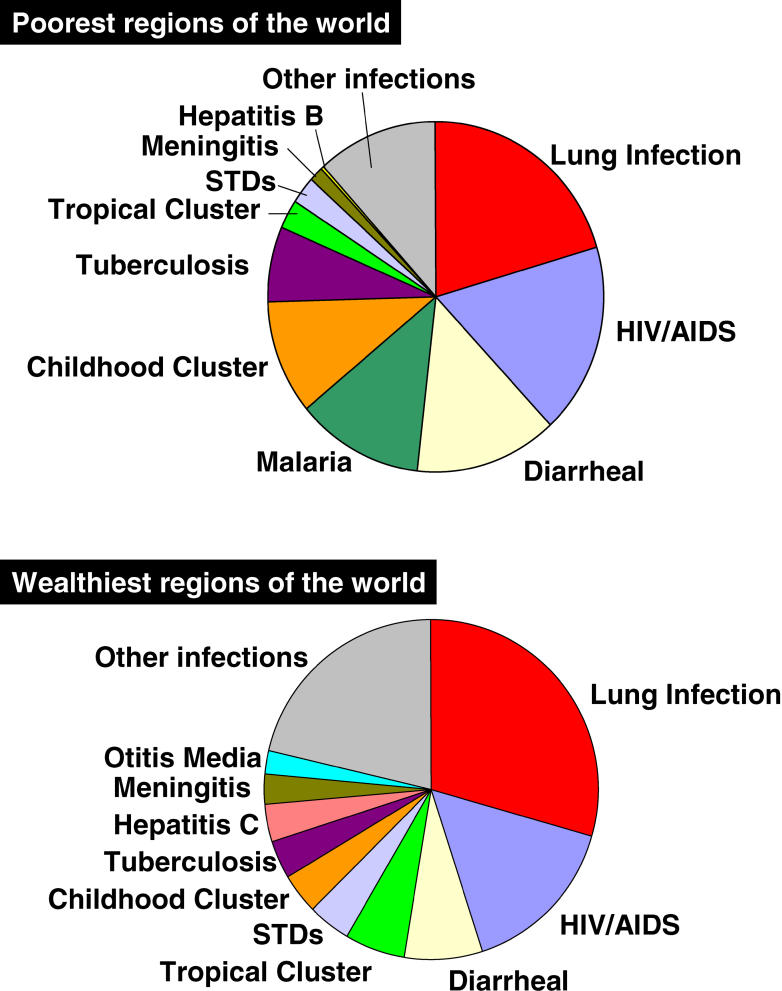
The Ten Leading Infections of the Poorest and Wealthiest Regions of the World These leading infections are represented as a fraction of the total infectious disease burden of that region, and are in clockwise descending order. (STDs, sexually transmitted deseases excluding HIV)

For those in wealthier populations, little further advancement is evident against lung infections. Focusing exclusively on the highest income populations (“Established Market Economies” or “High Income” groups in the WHO Burden of Disease Study), a comparison of the relative DALY losses due to lung infection shows little change from 1990 to 2002 (151 and 137 DALYs lost/100,000 population, respectively), whereas there have been dramatic improvements in the burdens due to other diseases within these wealthy communities. For example, among the wealthiest populations, HIV in 2002 caused less than half the disease it did in 1990 (from 159 to 72 DALYs lost/100,000 population). There are no DALY statistics available prior to 1990, but other indicators such as the US mortality rate due to pneumonia and influenza suggest that, for wealthy countries, there has been little or no progress against lung infections since the first half of the last century [6].

For several reasons, lung infections in wealthy countries seem poised to become even more of a concern in the near future. First, in these populations, advancing age makes lung infections increasingly more prevalent and life-threatening [7]. Therefore, demographic shifts resulting in an expanding elderly community within wealthy countries [8] suggest that more and more people are likely to suffer severely from lung infections. Second, novel respiratory infections emerge frequently, some of which can be highly virulent. Recent examples include SARS [9] and avian influenza [10]. If and when these new infections emerge, globalization increases the likelihood that such respiratory infections will become rapidly widespread [11]. Third, microbes that cause lung infections are increasingly resistant to previously effective antibiotics. While effective medical and public health practice will hopefully prevent the arrival of a “post-antibiotic” era [12], the continuously diminishing number of drugs effective against Streptococcus pneumoniae, Pseudomonas aeruginosa, and other common agents of community and hospital-acquired pneumonias raise concern.

## Efforts and Resources Marshalled against Lung Infections

All diseases included in Figures 1 and 2, and many not listed, are critical targets of research and health care. All require more funding and more effort than they now receive. However, if some diseases (such as lung infections) are less widely recognized as critical threats to our health, then resources and efforts will be allocated suboptimally, resulting in poorly tailored responses to public health needs.

Determining whether funds are contributing to research against a given disease is horribly inexact. Furthermore, the conceptual advances with most promise against a particular disease may more likely result from basic research than from disease-focused research. However, substantial resources are allocated to understanding and fighting particular diseases, and biomedical progress against those diseases is influenced by these targeted efforts. While the greatest burdens of disease and the greatest threats to the public health might be presumed to receive the greatest shares of research funding, they do not.

The US National Institutes of Health (NIH) spent approximately US$28 billion on health-related research in 2004 [13], of which US$287 million was allocated to lung infections. This is substantive and laudable, but it must be considered in perspective. It pales in comparison with the US$1.63 billion spent on biodefense. More NIH money is spent on smallpox research (US$324 million) alone than on lung infection research. While it is essential to be proactive in recognizing, preventing, and preparing for looming or emerging threats to public health, it may be questioned whether funding for speculated risks should so overwhelm funding for diseases already causing such tremendous burdens.

Lung infection research is also poorly funded when compared with other currently significant public health concerns. For example, US$2.85 billion were spent on HIV/ AIDS research, which is substantively improving prospects against this very important disease. It is remarkable, though, that lung infections cause a comparable or greater disease burden (Figures 1 and 2), yet they receive only one-tenth of HIV/AIDS research funding. In a similar vein, the NIH allocated comparable resources to lung infections as to sexually transmitted diseases (US$237 million), even though in wealthy countries such as the US lung infections cause seven times more disease than do sexually transmitted diseases (Figure 2), with even larger differentials in poorer countries. These figures from the NIH are but a few examples demonstrating that lung infections are relatively under-represented.

Reacting to the pandemic threat of the recently emerging avian influenza virus (H5N1), the president of the US recently requested a lump sum totaling US$7.1 billion [14]. The majority of requested funds in the president's plan, more than US$5.3 billion, would be slated for the manufacture, purchase, and stockpile of vaccines and antivirals targeting influenza. An additional US$0.8 billion would be allocated for research on new vaccines and antivirals against influenza, US$0.6 billion for influenza preparedness planning, and US$0.3 billion to help countries detect and contain influenza outbreaks. It is this author's opinion that the immediate need for such immense resources results from the potential of a severe influenza pandemic combined with many years of inadequate attention to lung infections. As of the time of writing this essay, the US Congress has yet to approve funding, and it remains unclear how much will be approved and how it will be deployed if approved, but a discrete set of funds may soon become available for fighting influenza specifically.

It is more difficult to assess resources distributed by private organizations, but it is again evident that lung infections are underemphasized. *U.S. News and World Report* identifies 20 charities as the largest to deal specifically with diseases and disease-related research [15]. Of these 20 leading charities, nine focus on cancer, two on organs (heart or kidney), two on classes of disease (mental illness or birth defects), and the rest on six specific diseases (muscular dystrophy, diabetes, multiple sclerosis, cystic fibrosis, Alzheimer disease, and arthritis). Perhaps the most prominent philanthropy focused specifically on infectious disease is The Global Fund to Fight AIDS, Tuberculosis, and Malaria [16]. This fund reports that it has attracted commitments of US$4.7 billion from national governments, private companies, and other contributors for fighting these three specific diseases. Such philanthropies perform wonderful services in improving health. Lung infections would similarly benefit from such a major philanthropic focus.

## Infectious Disease through the Prism of Microbiology

Why does the consistent burden of lung infections receive so little attention? It may result in part from our tendency to view infectious diseases from a microbiology perspective. Microbes can reasonably be portrayed and perceived as enemies to be attacked and defeated. Smallpox eradication is a powerful illustration of the potential of such an approach. Because AIDS is caused by HIV, malaria by *Plasmodia*, tuberculosis by *Mycobacteria*, and so on, defeating HIV and *Plasmodia* and *Mycobacteria* are widely recognized as valid goals in virtually any informed community.

While the idea of fighting against a specific microbe is attractive, and such an “us-against-them” mentality is effective at mobilizing commitments, a microbe-specific focus is appropriate for some infectious diseases more than others. Lung infections do not result from one or a few extremely virulent microbes especially adapted to living in our lungs. Rather, a tremendous variety of microbes causes lung infections, and a strategy focused on the microbes is destined to be a game of catch-up. The physiology of breathing requires our lungs to be enormously exposed to microbes, both from the external environment (the air) and from a microbe-rich part of our own anatomy (our upper airways). It is inevitable that microbes land in our lungs. In part for these reasons, respiratory infections are common, and new respiratory pathogens are likely to emerge frequently. Eliminating microbes in the lungs requires inflammatory responses that by their very nature compromise ventilation and blood-gas exchange. Thus, eliminating infection threatens physiology, and in part for this reason respiratory infections are often severe.

Tools are available for targeting some microbes causing lung infections (e.g., vaccines and antibiotics). Further research into reactive strategies directed specifically against individual microbes will likely improve our abilities to prevent or cure select lung infections. Few and marginally effective tools are available for targeting exposures or host responses to lung infection (e.g., ultraviolet germicidal irradiation or corticosteroids, respectively). Forward-thinking strategies might be directed at determinants of respiratory tract exposure, innate immune defenses against microbes in the lungs, and inflammatory injury resulting from lung infection. Advances in these areas will provide opportunities both to combat ongoing public health crises and to limit the potential threat from emerging pathogens.

## Conclusion

Like the proverbial elephant in the room, lung infections are a persistent problem not receiving the attention required. This may result in part from the nature of a disease lacking a single clear etiologic agent identified as a microbiological enemy. Whatever the reasons, it means that an important cause of human suffering is relatively underserved. Because the burden of disease is so substantial, greater efforts designed to elucidate the biology of lung infections, to generate novel therapeutic or prophylactic strategies, and to better deliver interventions to needy populations have the potential for tremendous public health impact.

## Abbreviations

DALY: disability-adjusted life year
ICD: International Classification of Diseases
SARS: severe acute respiratory syndrome
WHO: World Health Organization
